# Role of lactylation-induced macrophage failed phenotypic switching in sustaining inflammation of diabetic wounds

**DOI:** 10.3389/fimmu.2026.1777272

**Published:** 2026-03-06

**Authors:** Jiatong Wang, Kairui Wang, Zhihan Hu, Yunwei Wang, Yuchen Kang, Xiaohui Liu, Yuheng Zhang, Yi Liu

**Affiliations:** 1 Department of Burn Plastic and Wound Repair Surgery, Lanzhou University Second Hospital, Lanzhou, Gansu, China; 2 Department of oncology, Tangdu Hospital, Fourth Military Medical University, Xi’an, Shaanxi, China; 3 Key Laboratory of Stem Cells and Gene Drugs, Lanzhou, Gansu, China; 4 Department of orthopedics, Western Theater Air Force Hospital, Chengdu, Sichuan, China

**Keywords:** chronic inflammation, diabetic wounds, glycolysis, lactylation, macrophage

## Abstract

The impaired healing of diabetic wounds is closely associated with a persistent inflammatory response, wherein macrophages, as crucial immune effector cells in the local wound microenvironment, play a vital role in maintaining inflammatory equilibrium. Increasing evidence indicates that lactate, a product of glycolysis, is now recognized as a novel regulator of macrophage function by influencing gene transcription through protein lactylation on histone and non-histone substrates. This review seeks to outline the impact of chronic inflammation on macrophage phenotype (metabolism and polarization) and to clarify how subsequent protein lactylation alters macrophage biology, thereby impacting the progression of chronic inflammatory conditions such as diabetic wounds. These findings collectively provide new insights into the pathogenesis of impaired diabetic wound healing and underscore the potential of targeting protein lactylation as a therapeutic approach against chronic inflammation.

## 1 Introduction

Diabetes mellitus constitutes a significant global chronic disease with a continuously increasing incidence. Diabetic wounds, a prevalent complication, present a substantial clinical challenge due to their high frequency, persistent characteristics, and potential to result in amputation or death (1). The pathogenesis of these wounds is complex and involves multiple factors, including local metabolic disturbances, oxidative stress, and chronic inflammation. Central to this pathology is chronic inflammation, where macrophage dysfunction serves as a critical driver of the sustained and excessive inflammatory response.

Macrophages exhibit high plasticity, allowing them to adapt their phenotype and function in response to microenvironmental cues. This adaptability enables them to exert either pro-inflammatory or anti-inflammatory effects at various stages of wound healing. Consequently, regulating macrophage polarization to maintain local immune balance is essential for diabetic wound healing (2, 3). Recent studies demonstrate that epigenetic regulation is pivotal in controlling macrophage polarization and function (4), acting as a crucial molecular link between the dysregulated local microenvironment of diabetic wounds and persistent inflammation (5).

Lactylation, a novel form of epigenetic regulation, directly influences gene expression and cellular function. Its role in regulating macrophage polarization and biological functions emerges as a prominent focus in immunometabolism research (6). Traditionally, lactate is viewed merely as the end product of glycolysis. However, in-depth investigations into the “Warburg effect” progressively uncover its biological functions in non-tumor cells, particularly immune cells. Studies reveal that lactate serves not only as an energy substrate but also as a signaling molecule. It participates in key pathophysiological processes, including epigenetic regulation, immune responses, and angiogenesis, through protein lactylation (7–9).

This review begins with the diabetic wound microenvironment driving macrophage metabolic reprogramming toward aerobic glycolysis. Excessive lactate production/accumulation induces hyper-lactylation of histone/non-histone proteins to epigenetically reinforce pro-inflammatory gene expression while repressing repair, culminating macrophage defective M1/M2 polarization switching and sustaining chronic inflammation. It aims to enrich the pathogenesis of healing disorders in diabetic wounds and lay the groundwork for the development of novel treatment approaches focusing on lactylation and its principal regulatory factors.

## 2 The pathological cascade reaction driven by the diabetic wound microenvironment

Normal wound repair requires precise spatiotemporal regulation of its initiation and resolution. In diabetes, however, this repair cascade becomes markedly dysregulated. This dysregulation originates from the unique diabetic wound microenvironment, which is characterized by persistent hyperglycemia, ischemia/hypoxia, and chronic excessive inflammation. These pathological features interact with key cellular populations within the wound milieu, further exacerbating the impairment of healing.

### 2.1 Microenvironment abnormality in diabetic wounds

**Figure 1:**
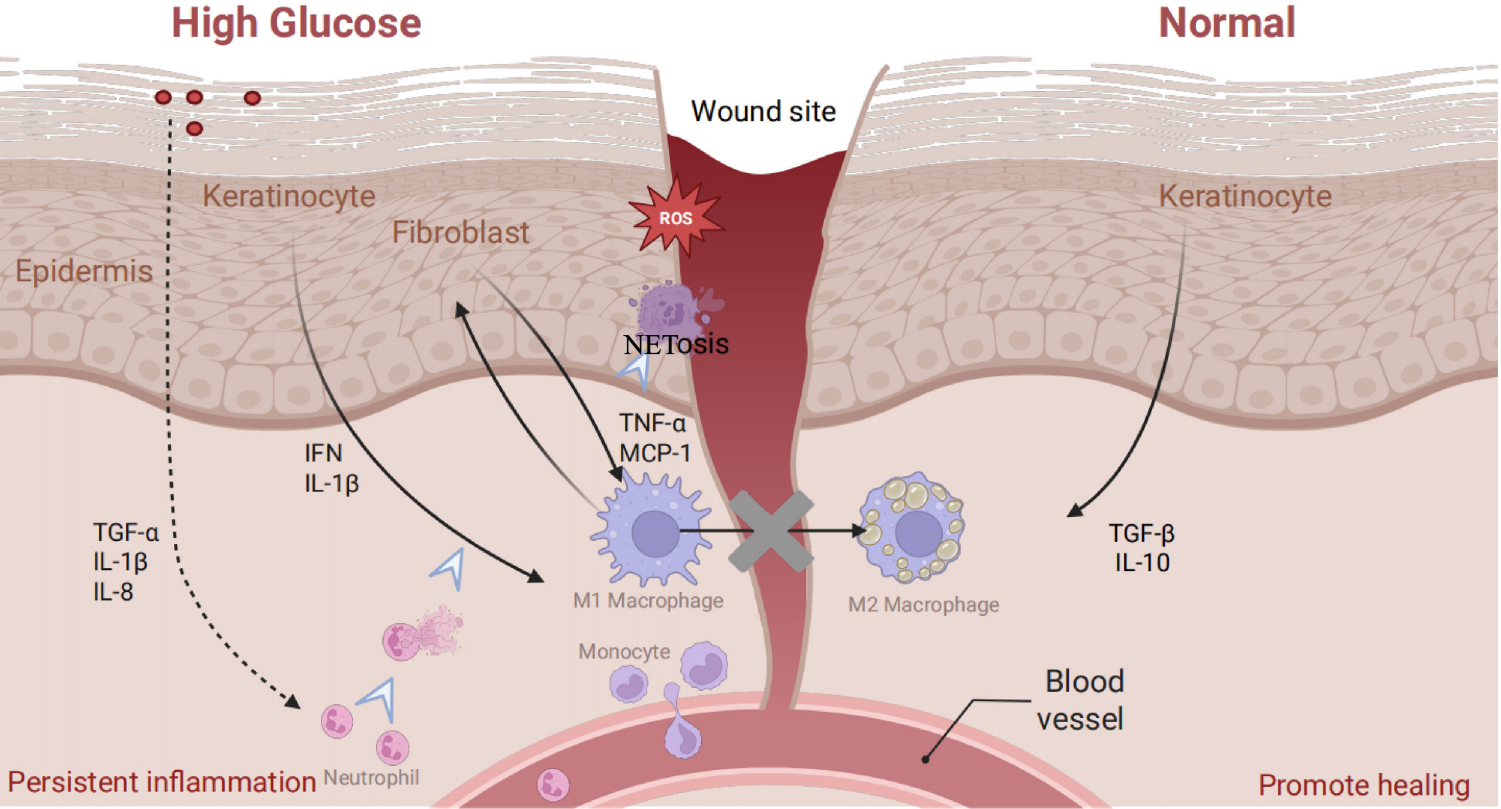
Characteristics of the immune microenvironment in normal and diabetic wounds.

When skin integrity is disrupted, immune cells, particularly macrophages, participate in all stages of wound repair (10) (hemostasis, inflammation, proliferation, and remodeling) (11, 12). During the initial phase of injury, tissue-resident macrophages (RTMs) become activated and migrate to the wound site in response to damage-associated molecular patterns (DAMPs). Perivascular RTMs promptly detect damage signals through purinergic receptors (13). These activated macrophages collaborate with platelets to generate various chemokines and cytokines, including interferons, to attract other immune cells (14). However, the diabetic environment alters this normative response (**Figure 1**). For instance, damaged oxidized mitochondrial DNA (Ox-mtDNA) released from injured cells under high glucose is engulfed by macrophages, triggering an inflammatory response via the cGAS-STING pathway. Ox-mtDNA activates cGAS, which produces cGAMP to activate STING, leading to type I interferon (IFN) production. Furthermore, non-immune cells promote this hostile environment. The hyperglycemic environment hinders keratinocyte migration and proliferation while increasing the secretion of pro-inflammatory factors such as TNF-α and IL-1β (15). Simultaneously, continuous Toll-like Receptor 4 (TLR4) signaling boosts MIP-2 and MCP-1 levels, creating a pro-inflammatory milieu (16, 17). Additionally, accumulated AGEs inhibit TGF-β release from keratinocytes. In fibroblasts, hyperglycemia diminishes synthetic capacity, reducing ECM deposition (18); their exosomes also fail to regulate macrophage polarization effectively (19). This multifaceted dysregulation creates an initial trigger characterized by severe immune stress and metabolic imbalance.

### 2.2 Macrophage metabolic reprogramming to aerobic glycolysis

A hallmark of diabetic wounds is macrophage-mediated immune dysregulation, which closely relates to altered energy metabolism. As research into the connections between immune regulation and disease mechanisms deepens, studies reveal that the immune functions of macrophages rely on specific and dynamically interconnected metabolic programs, with aerobic glycolysis playing a crucial role (20). Aerobic glycolysis describes the process in which cells preferentially metabolize glucose through the glycolytic pathway to produce lactate, even in the presence of sufficient oxygen, plays a critical role in cancers and non-cancerous diseases, such as chronic inflammatory diseases (21).

**Figure 2:**
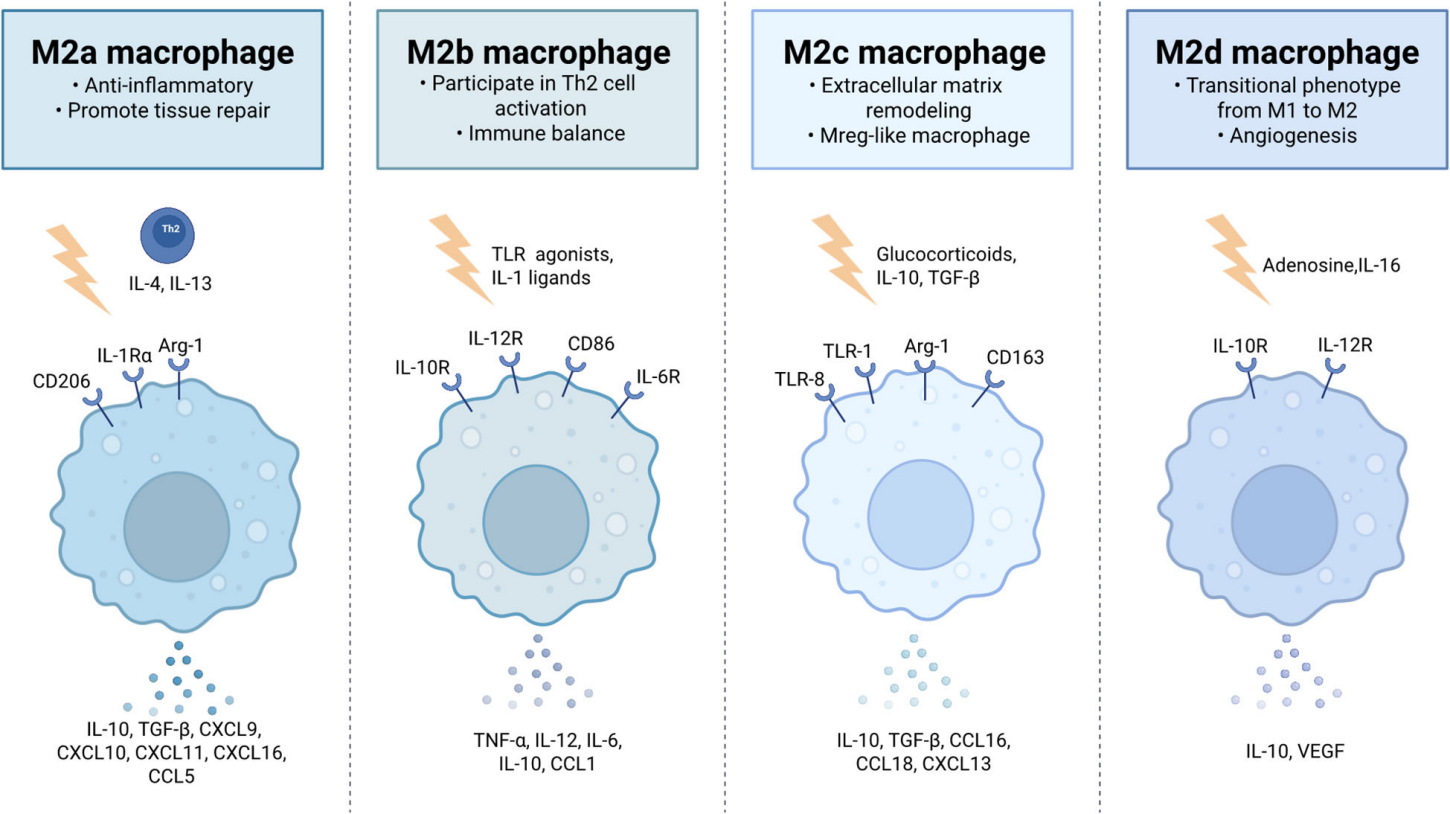
Subtypes of M2 macrophages.

Within the wound microenvironment, macrophages adapt their phenotype and function dynamically in response to local cytokine cues, a phenomenon known as macrophage polarization. The pro-inflammatory and anti-inflammatory polarization states represent the endpoints of a broad spectrum of activation (22, 23). Macrophage polarization represents a highly dynamic process, which can be broadly categorized into the pro-inflammatory M1 state and the anti-inflammatory M2 state. M1 macrophages primarily rely on glycolysis to produce reactive oxygen species (ROS) and nitric oxide (NO), while secreting pro-inflammatory factors such as TNF-α, IL-1β, IL-12, and IL-23, thereby mediating pro-inflammatory responses and microbial killing. In contrast, M2 macrophages mainly depend on fatty acid oxidation and the tricarboxylic acid (TCA) cycle for energy production, generating metabolites such as L-proline and polyamines. They secrete anti-inflammatory factors like IL-10 and TGF-β, promoting angiogenesis and tissue repair (24). As shown in **Figure 2**, M2 macrophages can be subdivided into four subsets based on gene expression profiles and functional characteristics. M2a macrophages, induced by IL-4/IL-13 from Th2 cells, express CD206, IL-1Ra, and IL-1RII, secrete IL-10 and TGF-β, and play a role in anti-inflammatory responses and tissue repair (25, 26). M2b macrophages, activated by TLR agonists or IL-1 receptor ligands, have immunoregulatory properties, co-produce IL-6, IL-10, IL-12, and TNF-α, and are involved in Th2 cell activation (27). M2c, also known as Mreg-like macrophages, are induced by glucocorticoids, IL-10, and TGF-β, express CD163 and scavenger receptors, secrete IL-10, TGF-β, and MMP9, and contribute to matrix remodeling (28, 29). M2d macrophages, induced by adenosine and IL-6, act as a transitional phenotype from M1 to M2, promoting angiogenesis through VEGF and IL-10 secretion, and exert anti-inflammatory effects by suppressing IL-12 and TNF-α expression (30, 31). This precise differentiation of macrophage subsets ensures a delicate balance between resolving inflammation and promoting tissue regeneration during wound healing.

Within the inflammatory microenvironment, infiltrating immune cells rely heavily on glycolysis to meet the high energy demands associated with rapid recruitment and phagocytosis, resulting in the production of substantial amounts of lactate. In diabetic wounds, persistent chronic inflammation leads to a significant immune imbalance in macrophages: M1 macrophages remain continuously activated and sustain elevated levels of aerobic glycolysis. Notably, CD40 signaling functions as a key metabolic switch by modulating the intracellular NAD+/NADH ratio, which guides cells in adaptively transitioning between metabolic pathways, including glycolysis, oxidative phosphorylation (OXPHOS), and fatty acid oxidation (32). Mitochondria, as the cellular energy centers, sense local microenvironmental signals and dynamically adjust energy metabolism to support both resident and recruited cells in inflammatory responses. Research indicates that various immune cells, including macrophages, dendritic cells, T cells, and neutrophils, undergo a metabolic shift from OXPHOS to glycolysis, and this metabolic reprogramming is crucial for immune regulation.

### 2.3 Lactate accumulation and metabolic processes

The production and functional reprogramming of lactate establish a critical link between metabolic and immune pathways. The metabolic reprogramming results in the production of substantial amounts of lactate, which is a crucial end product of glycolysis, binds to specific receptors on immune cells to regulate their metabolic state (7). Specifically, lactate dehydrogenase facilitates the bidirectional conversion between pyruvate and lactate, as well as the NAD+/NADH redox cycle (33). In biological systems, lactate exists as a protonated molecule in acidic microenvironments or as a lactate ion at physiological pH (34). Lactate could regulate energy transfer and cellular communication via intracellular (cytosol-mitochondria/peroxisome) and intercellular shuttles, critical for maintaining its intra-/extracellular homeostasis (35, 36). Its transport primarily involves monocarboxylate transporters (MCTs), which mediated transport direction depends on lactate/proton gradients; their coordination enables shuttling between glycolytic and OXPHOS cells, key to tissue lactate homeostasis (37). MCT1, widely expressed and high-affinity, mediates bidirectional transport; in macrophages, it promotes inflammation via HIF-1α-upregulated PFKFB3 (38). MCT4, expressed in highly glycolytic tissues, functions as a unidirectional exporter, with its shuttling among inflammatory cells potentially exacerbating inflammation via NF-κB activation (39). Consequently, in the diabetic wound context, the sustained elevated levels of aerobic glycolysis result in considerable lactate accumulation. This accumulated lactate serves not only as a metabolic byproduct but also as an important signaling molecule that influences various biological processes, including the regulation of energy metabolism, modulation of inflammatory responses, and neuroprotection. It signals via the G protein-coupled receptor GPR81 (HCA1), GPR81 exhibits a broad response range: low concentrations initiate partial signaling, while higher concentrations enhance activation (40), to regulate inflammatory responses and lipid metabolism (41–43). Metabolically, lactate is converted to pyruvate or generates lactyl-coenzyme A (lactyl-CoA), establishing a cross-regulatory link between energy metabolism and epigenetics.

## 3 Lactate mediated epigenetic regulation and dynamic transcriptional imbalance

Macrophage metabolic reprogramming in diabetic wounds leads to pathological overaccumulation of lactate, which, via lactic acidification, converts metabolic stress into epigenetic signals that can stably alter chromatin structure and protein functions. Lactic acidification exerts dual regulatory effects of “pro-inflammation” and “pro-repair” on macrophages, but in the context of diabetic sustained high lactic acid environment, “hyper-lactylation” leads to severe tilt of this balance towards pro-inflammation, resulting in transcriptional dysregulation in diabetes and aggravating healing defects.

### 3.1 Lactylation modification of histones and non-histones

The accumulated lactate serves as a substrate for histone lactylation, a recently identified epigenetic modification. This modification regulates chromatin structure and gene expression by altering histone lysine residues. Mammals produce two lactate enantiomers: L-lactate and D-lactate, with L-lactate predominating. As the end product of glycolysis, it is mainly produced endogenously and can be considered as an “alarm signal” of diabetic metabolic disorders and a role in various physiological processes, including proteins lysine lactylation (Kla) modification (44), covalently attaching to histone lysine residues in a concentration-dependent manner (45). The most prevalent lactylation site is histone H3 lysine 18 lactylation (H3K18la), which can significantly impact histone function and cellular regulatory processes (46). In contrast, D-lactate mostly originates from the fermentation of undigested carbohydrates by the intestinal microbiota, which may indicate dysbiosis of the intestinal microbiota (44), and there is evidence that it is associated with diabetic peripheral neuropathy (47).

Apart from nuclear histones, lactylation modifications also extend to non-histone proteins in various cellular locations like the cytoplasm, influencing a wide range of cellular functions (48). Lactylation modifications have been detected in various proteins within the macrophage nucleus, cytoplasm, mitochondria, endoplasmic reticulum, and cell membrane (49, 50). This mechanism transforms the biochemical signal of lactate accumulation into a tangible molecular alteration in the macrophage proteome. Current research on lactylation primarily focuses on histone lactylation in macrophages and its associated diseases. Although these findings highlight the significant impact of lactylation as a post-translational modification, the specific enzymes regulating lactylation and their molecular mechanisms remain unclear, representing an important direction for future research.

### 3.2 Dual impact of lactylation on macrophages

**Figure 3:**
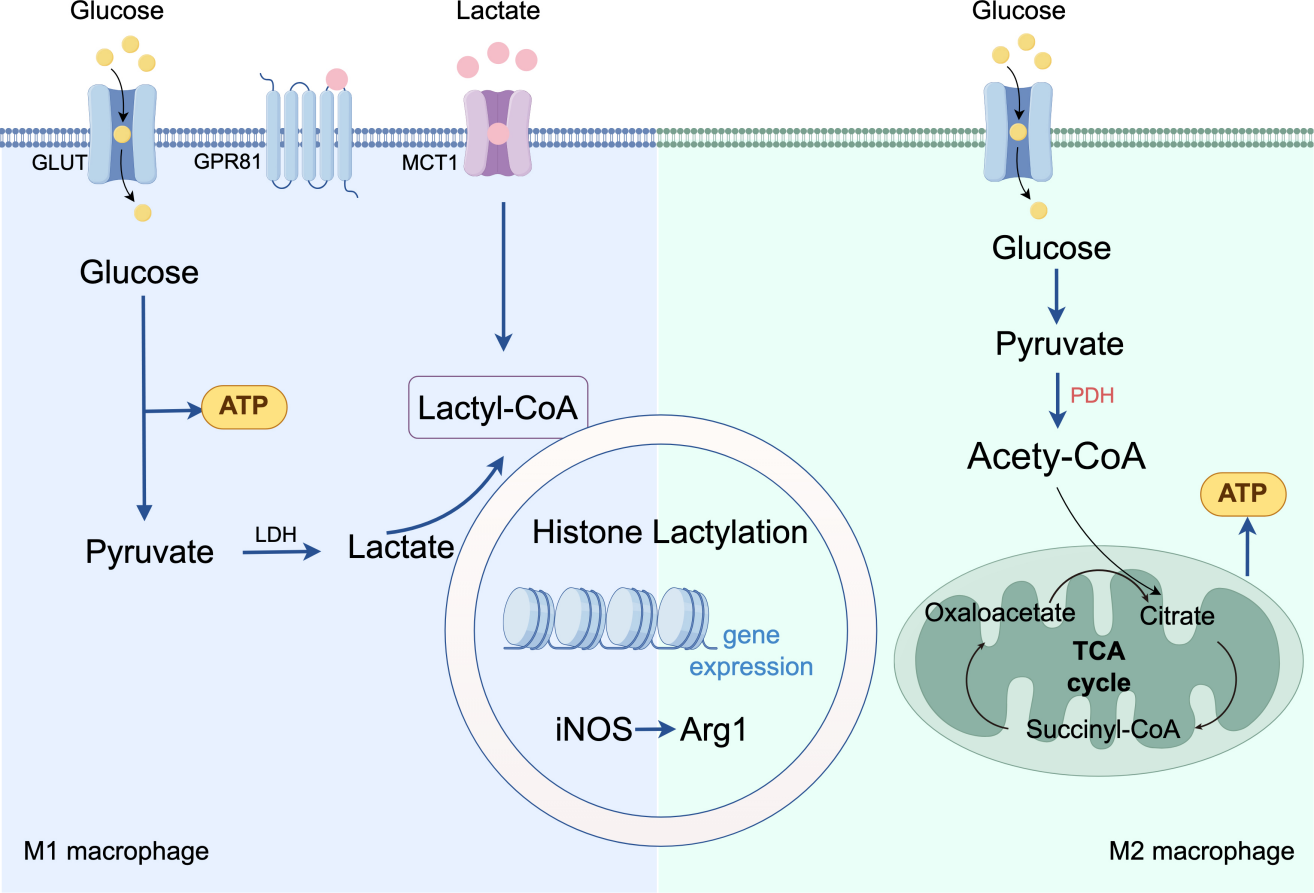
Mechanism of lactylation-mediated regulation of macrophage polarization.

Widespread lactylation leads to complex transcriptional outcomes, exerts a dual effect on macrophages (**Figure 3**). During the early inflammatory phase, lactate synergizes with other pro-inflammatory factors to upregulate the expression of pro-inflammatory genes (51). Over time, the “lactate clock” initiates: lactylation modifications launch the regulation of gene transcription and trigger the transition of macrophages to the M2 phenotype in a time-dependent manner—specifically, studies demonstrate that at 16–24 hours, lactate activates the promoter regions of M2 signature genes, such as Arg-1, through H3K18la-this transition subsequently alleviates inflammatory damage and promotes tissue repair (6). However, in the context of prolonged metabolic stress or sustained chronic inflammation typical of diabetic wounds, this temporal regulatory mechanism is disrupted. While histone lactylation promotes the transition of macrophages from M1 to M2 under normal repair conditions, in the context of persistent inflammation, it can exacerbate inflammatory responses. The diabetic microenvironment locks lactate modification into a pro-inflammatory phenotype by enzyme activity disorders and substrate selection shift, forming a cascade of “metabolic disorders to epigenetic abnormality to immune imbalance”. Dysregulated lactylation allows for the epigenetic sustenance of a pro-inflammatory state rather than a timely transition to repair, regulating the expression of genes related to inflammation, cytokines, and metabolic pathways (42, 43, 52). In many chronic inflammation-related diseases, the shift in macrophage polarization profoundly influences disease progression and outcomes, but the specific mechanisms still require clarification through more in-depth fundamental research.

## 4 The vicious cycle of macrophage polarization arrest and chronic inflammation

In the diabetic wound microenvironment, high levels of lactate may further solid the polarized stagnation state of macrophages by driving abnormal “hyper-lactylation” modification. This “metabolic-phenotype” vicious cycle—where metabolic products (lactate) exacerbate phenotypic arrest, and the arrested phenotype (M1) continuously generates more metabolic products—eventually leads to unresolving chronic, severely hindering the wound repair process.

### 4.1 Macrophage polarization arrest and dysfunction

Transcriptional dysregulation caused by lactylation modification manifests as severe phenotypic dysfunction in macrophages. During normal wound healing, the newly recruited monocytes quickly react to signals in the wound microenvironment, such as cellular debris, hypoxia, microbial products, and activated lymphocytes, and differentiate into M1 macrophages, exert pro-inflammatory effects in the early phase (days 1-3), under the influence of factors like IFN-γ, LPS, GM-CSF and TNF. Key transcription factors involved in this process comprise NF-κB, the STAT family (STAT1/2/4/5), and the IRF family (IRF1/3/5/8) (53–56). The activated M1 macrophages, through their pattern recognition receptors (PRRs), identify both pathogen-associated molecular patterns (PAMPs) and DAMPs. They form phagolysosomes, release antimicrobial substances such as reactive oxygen and nitrogen species, and efficiently eliminate cellular debris (57–59). To facilitate the transition from the inflammatory to the proliferative phase, macrophages gradually shift to an M2-dominant phenotype in response to microenvironmental signals, which peak around day 7 (60–62). This phenotypic switch accompanies functional and metabolic reprogramming, including the downregulation of mitochondrial ROS levels and adaptive metabolic changes.

In diabetes, hyperglycemia impedes this M1-to-M2 transition through multiple mechanisms. In the early inflammatory stage, hyperglycemia suppresses chemokine expression, delays monocyte recruitment to the wound, and impairs neutrophil clearance, which exacerbates oxidative stress. Concurrently, it upregulates the expression of pro-inflammatory factors such as TNF-α, IL-1, and IL-6 through epigenetic regulation, promoting M1 polarization. It also affects bone marrow hematopoietic stem cells, increasing circulating Ly6C-high monocytes that infiltrate the wound and differentiate into M1 macrophages (63).

### 4.2 Self-enhancing circuit of inflammatory microenvironment

Crucially, hyperglycemia impairs macrophage efferocytosis, the clearance of apoptotic neutrophils, which is a key step for M1/M2 switching (64, 65). Because the clearance of apoptotic neutrophils is obstructed, their released toxic components activate the NLRP3 inflammasome via the TLR-4/TLR-9/NF-κB and ROS/TXNIP pathways, which further amplifies inflammation (66). Additionally, hyperglycemia induces macrophage senescence, characterized by a senescence-associated secretory phenotype (SASP) and reduced plasticity (67). Consequently, M1 macrophages assume a dominant role, maintaining high expression levels of pro-inflammatory factors like TNF-α, IL-1β, and IL-6 (53, 68–72), while the M2-dominant phenotype required for the proliferative phase suppresses inflammation and promotes angiogenesis fails to manifest effectively (73, 74).

Ultimately, this cascade of dysfunction traps the diabetic wound in a “chronic inflammation–impaired repair” pathological cycle. The core mechanism underlying impaired diabetic wound healing resides in the stagnation of the repair process due to immune dysregulation. Healing abnormally stagnates in the inflammatory phase and fails to progress to the proliferation phase. Specific outcomes of this stagnation include the creation of a cascading amplification of inflammation. TNF-α from M1 macrophages prompts fibroblasts to release MCP-1, attracting more macrophages and worsening local inflammation. This persistent inflammation impairs fibroblast function, establishing a detrimental cycle (75). Prolonged pathway activation polarizes macrophages toward a pro-inflammatory M1 phenotype and intensifies the inflammatory response, perpetuating chronic inflammation in the wound (76). Thus, the imbalance of the immune microenvironment is crucial for sustaining chronic excessive inflammation (22), leading to the final clinical presentation of non-healing diabetic wounds. The dysfunctional macrophage phenotypic switch represents a pivotal driver of chronic inflammation and impaired healing in diabetic wounds, thus necessitating further mechanistic investigation and the development of targeted therapies.

## 5 Mechanisms of chronic inflammatory diseases influenced by macrophage lactylation

**Figure 4:**
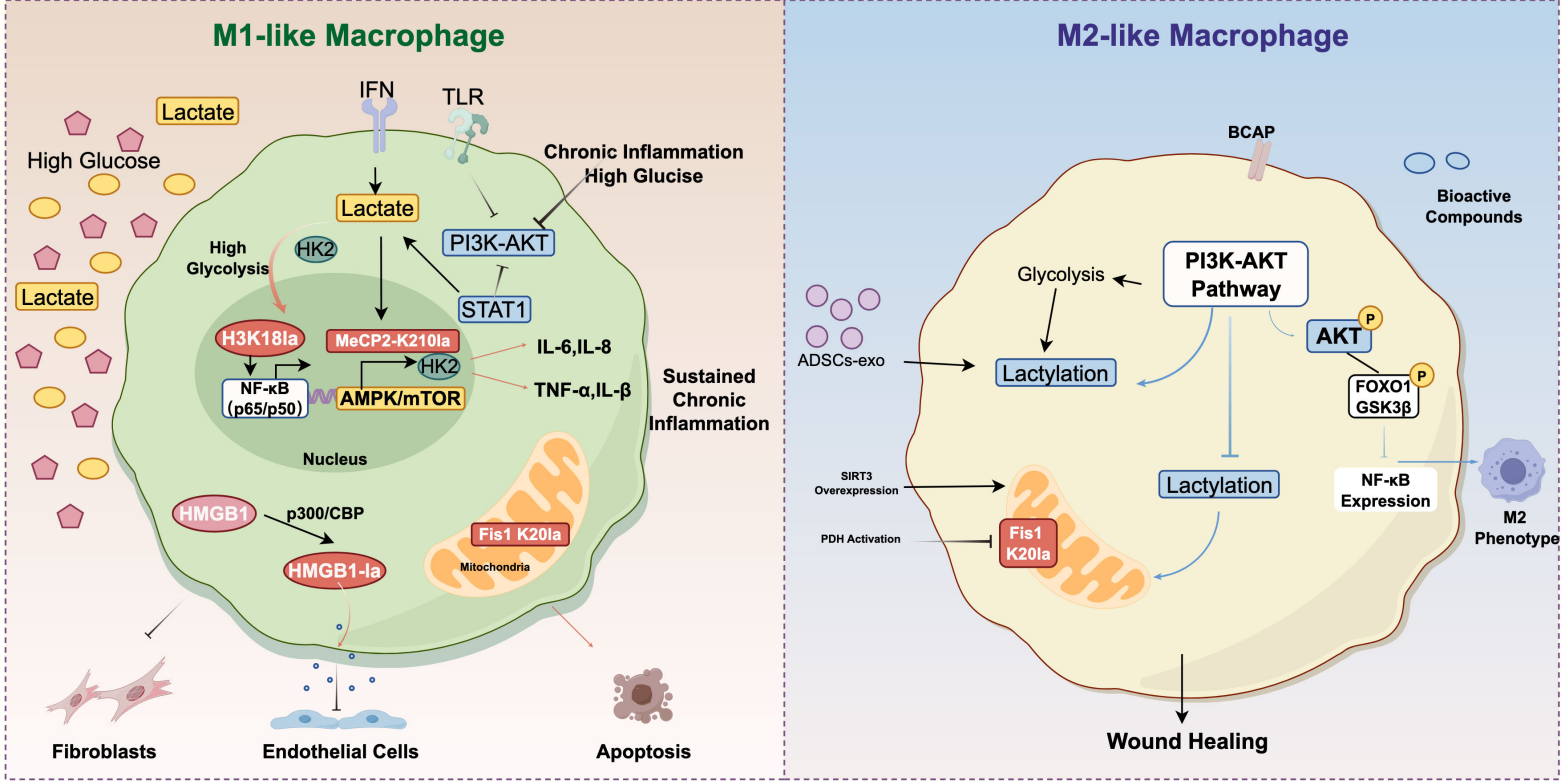
Mechanisms of chronic inflammatory diseases influenced by macrophage lactylation.

Inflammation is a defensive response of the immune system against inflammatory stimuli. Moderate inflammation, mediated by immune cells, serves as a protective mechanism to eliminate pathogens and repair damaged tissues (77). However, excessive or persistent inflammation disrupts homeostasis and leads to chronic inflammatory diseases, such as diabetic complications, inflammatory bowel disease, sepsis, and pulmonary fibrosis (**Figure 4**). Research indicates that a hallmark of the inflammatory microenvironment is significantly elevated lactate levels, which mediate functional changes in immune cells through lactylation modification (78, 79). Macrophages, as key participants in the pathogenesis of various inflammatory diseases, exhibit high plasticity and coordinate immune responses by undergoing phenotypic transitions (including homeostatic, pro-inflammatory, pro-fibrotic, and reparative phenotypes) in response to diverse microenvironmental signals (80–83). In diabetic wounds, excessive lactate accumulation induces “hyper-lactylation,” activating interleukin-6 (IL-6) production and triggering the IL-6 receptor/Stat3 signaling pathway to exacerbate inflammation (84, 85). This aberrant metabolic state synergizes with hypoxia and chronic inflammation, creating a self-reinforcing vicious cycle that impairs wound healing. Furthermore, metabolic reprogramming centered on hypoxia, enhanced glycolysis, and lactylation plays a critical role in regulating key signaling pathways governing wound repair and immune responses in diabetic wounds, contributing to their non-healing condition (86).

### 5.1 By regulating NF-κB signal pathway

The NF-κB pathway is a canonical pathway regulating macrophage polarization, involved in inflammatory and immune responses. In macrophages, signals such as IFN and TLR drive pro-inflammatory M1 polarization via the NF-κB/STAT axis, particularly through STAT1. Recent studies have shown that high lactate levels in natural aging and Alzheimer’s disease models promote H3K18la in microglia and hippocampal tissue. This modification directly activates the NF-κB pathway by enhancing binding to the promoters of Rela (p65) and NFκB1 (p50), thereby upregulating senescence-associated factors (e.g., IL-6, IL-8) and promoting the release of pro-inflammatory cytokines (e.g., TNF-α, IL-1β) to exacerbate chronic inflammation (87, 88). Metabolic reprogramming of macrophages in diabetic wounds causes immune microenvironment dysregulation accompanied by impaired M1-to-M2 transition; lactate accumulated in this process further activates pro-inflammatory signaling pathways (e.g., NF-κB, STAT1) in M1 macrophages (89), inhibiting repair, exacerbating polarization arrest, and trapping the wound in persistent chronic inflammation. Additionally, the hyperglycemic wound environment induces hyperactive glycolysis in fibroblasts and endothelial cells, inhibiting collagen secretion and vascularization, thus delaying wound repair at multiple levels (86). Therefore, targeting the NF-κB/STAT axis to disrupt lactate-driven epigenetic dysregulation presents a promising therapeutic strategy to rescue M1/M2 polarization imbalance in chronic inflammation like diabetic wounds.

### 5.2 By regulating PI3K-AKT signal pathway

The PI3K-AKT pathway is a key pathway for angiogenesis and tissue repair, and also plays a critical role in regulating macrophage survival, proliferation, migration, and polarization (90). This pathway exerts a negative regulatory effect on TLR and NF-κB signaling in macrophages (91): activation of PI3K or AKT kinases attenuates LPS stimulation of macrophage TLRs, whereas inhibiting PI3K in TLR-activated macrophages enhances NF-κB activation and iNOS expression, thereby activating pro-inflammatory signaling pathways (92). Under physiological conditions, highly expressed B Cell Adaptor for Phosphoinositide 3-Kinase (BCAP) in macrophages activates the PI3K-AKT pathway, driving aerobic glycolysis in M1 macrophages, inducing histone lactylation, promoting phosphorylation and activation of AKT downstream targets FOXO1 and GSK3β, inhibiting NF-κB expression, and ultimately driving M1-to-M2 macrophage polarization (81). However, in diabetic wounds, hyperglycemic and persistently inflammatory environments inhibit normal PI3K-AKT activation, impairing angiogenesis and the M1-to-M2 transition (93, 94). This phenomenon may relate to aberrant histone lactylation induced by excessive lactate accumulation from persistent inflammation, but the specific mechanism remains unclear. To address this, adipose-derived stem cell exosomes (ADSCs-exo) and other bioactive compounds have shown potential to promote wound repair by targeting PI3K-AKT activation (93). In the future, engineered modification of ADSCs-exo via advanced molecular techniques (e.g., gene editing, synthetic biology) is expected to further enhance their therapeutic efficacy, offering new directions for developing novel strategies against persistent inflammation in diabetic wounds.

### 5.3 By regulating other related mechanisms

Sepsis, similar to diabetic wounds, exhibits characteristic metabolic disturbances marked by significantly elevated lactate levels. Differential lactylation patterns between patients and healthy individuals may lead to contrasting outcomes. In septic patients, H3K18la and lactylation of high mobility group box 1 (HMGB1) are markedly increased in tissues, correlating positively with infection severity (95). Mechanistically, lactate activates p300/CBP to induce HMGB1 lactylation in macrophages, which, upon exosomes release, downregulates endothelial tight junction proteins, disrupting vascular barrier function (49). Furthermore, serum HMGB1 levels are significantly elevated in diabetic wound patients and correlate with vascular endothelial dysfunction markers (96). Hyperactive HMGB1 activates downstream TLR4/NF-κB signaling, exacerbating local inflammation and impairing wound healing. Studies have demonstrated that in septic patients with blood lactate ≥4 mmol/L, the 20th lysine residue of mitochondrial fission protein 1 (Fis1 K20la) undergoes lactylation, promoting excessive mitochondrial fission, ATP depletion, mtROS burst, and mitochondrial apoptosis pathway activation (97). Targeting pyruvate dehydrogenase E1 activation or SIRT3 overexpression to reduce Fis1 K20la improves mitochondrial function, attenuates inflammation, and corrects microcirculatory defects, offering a metabolic remodeling-based therapeutic strategy for diabetic wounds.

Proteomic studies have revealed that in neuroinflammation, methyl-CpG-binding protein 2 (MeCP2) in microglia undergoes lactylation modification at the lysine 210 (K210) site, exerting a critical metabolic-epigenetic regulatory role: On one hand, it activates transcription of glycolytic/inflammatory genes (notably hexokinase 2, HK2), exacerbating lactate accumulation; on the other hand, it impairs mitochondrial respiration and disrupts metabolic signaling pathways (e.g., AMPK/mTOR), collectively maintaining chronic neuroinflammation (98). Elimination of MeCP2-K210 lactylation via K210R mutation or inhibitors effectively suppresses glycolysis, restores mitochondrial function, and attenuates inflammation. These findings establish MeCP2-K210 lactylation as a key switch regulating tissue-resident macrophage polarization via the HK2/mTOR axis, revealing its potential as a therapeutic target.

The key evidence mentioned above has been organized in **Table 1**, including the disease/model complex, type of research, lactylation sites, and the resulting biological functions.

**Table 1:** Lactylation contexts, sites and biological functions.

Model	Type of research	Target of lactylation	Biological function following lactylation	Reference
Bone marrow derived macrophage	*in vitro*/vivo	H3K18	Upregulated IL-6/STAT3 expression	Dichtl S et al. (2021) (84)
Alzheimer’s disease (microglia)	*in vitro*/vivo	H3K18	Activation of NF-κB signaling pathway	Wei L et al. (2023) (87)
Alzheimer’s disease (microglia)	*in vitro*/vivo	H4K12	Glycolysis/H4K12la/PKM2 positive feedback	Pan RY et al. (2022) (88)
Neovascular age-related macular degeneration (macrophages/microglia)	*in vitro*/vivo	/	Activation of HIF-1α/2α, NF-κB signaling pathway	Liu Z et al. (2022) (89)
Diabetic foot ulcers (macrophages)	bioinformatics analysis	/	Activation of TNFα signaling pathway	Hu B et al. (2025) (86)
Septic shock (macrophages)	*in vitro*	H3K18	Upregulated IL-6 expression	Chu X et al. (2021) (95)
Sepsis (macrophages)	*in vitro*/vivo	HMGB1	Increased endothelium permeability	Yang K et al. (2022) (49)
Acute kidney injury (renal tubular epithelial cells)	*in vitro*/vivo	Fis1 K20	Increased excessive mitochondrial fission	An S et al. (2023) (97)
Neuroinflammation (microglia)	*in vitro*/vivo	MeCP2-K210	Upregulated glycolysis/inflammatory genes expression; impaired mitochondrial respiration; disrupted metabolic signaling pathways	Zhang Z et al. (2025) (98)

## 6 Discussion

Macrophages, characterized by their high plasticity, serve as crucial immunomodulatory cells in diabetic wound healing. Research indicates that immune dysregulation, resulting from dysfunctional macrophages in the diabetic wound microenvironment, constitutes a critical factor contributing to sustained chronic inflammation and impaired healing (99). Lactylation, initially identified as a post-translational modification on macrophage histones, occurs through lactate, a product of glycolysis. This finding illustrates the presence of metabolic-epigenetic crosstalk between the metabolic profile and phenotypic switching of macrophages (100). This article systematically elucidates the relationship between chronic inflammation and macrophage dysfunction in diabetic wounds, the role of lactate and lactylation in macrophage polarization, and outlines the molecular mechanisms through which lactylation regulates macrophage involvement in the progression of chronic inflammatory diseases.

Although researchers extensively study the role of macrophage metabolic reprogramming in disease progression, investigations into the mechanisms by which lactylation regulates macrophage plasticity remain in their infancy. The endogenous “lactate clock” in macrophages highlights the critical role of histone lactylation in mediating the transition from M1 to M2 macrophages (101). The regulatory mechanisms involved include the generation of lactyl-coenzyme A through enzymatic reactions, facilitated by both endogenous lactates produced by aerobic glycolysis in M1 macrophages and exogenous lactate taken up via MCTs. Catalyzed by the acetyltransferase p300, lactate is added to the lysine tails of histones in a p53-dependent manner. However, several key scientific questions remain unresolved in this process (102). First, the dynamic differences in lactate concentration resulting from metabolic shifts in macrophages within the diabetic versus normal wound microenvironment and their pathological significance require further exploration. Second, the lactate concentration threshold that initiates the “lactate clock” and the differential binding of lactylation modifications to specific amino acid sites, mediated by varying lactate concentrations, need clarification. Third, the precise regulation of key enzymes involved in this stage, such as lactate dehydrogenase A (LDHA), LDHB, MCT1, MCT4, and lactyl-CoA, warrants investigation.

Based on the exploration of the mechanism of lactic acidification-regulating macrophages participating in chronic inflammation of diabetic wounds, macrophage metabolism and epigenetics could be a promising therapeutic avenue. The primary strategies are to modulate the local lactic acid level, by inhibiting glycolysis (e.g., targeting HK2), activating oxidative metabolism (e.g. using DCA), or regulating MCT transporters, etc., from the upstream to relieve the driving factors. Second is precision epigenetic intervention, directly using inhibitors lactic acid transferase (e.g., p300/CBP) or activators of de-lactic acidification enzyme (e.g., SIRT3), etc., to regulate the lactic acidification modification itself. Third is key pathway repositioning, by drug means to re-activate the damaged repairment (e.g., activating the PI3K/AKT axis by growth factors or ADSCs-exo) or to inhibit the hyperactive inflammatory pathway (e.g., NF-κB/STAT1 axis), etc., to correct the downstream functional defects. In addition, the most targeted strategy may be to develop interventions against lactic acidification proteins with specific dysfunction (e.g., blocking peptide, site editing).

Finally, the specific role of lactylation-mediated post-translational modifications in diabetic wound healing remains to be elucidated. Investigating the molecular mechanisms that elucidate the specific machinery through which histone lactylation regulates the “lactate clock” in diabetic wounds is essential. The focus of mechanistic research centers on modulating lactate levels within the diabetic wound microenvironment. This approach aims to decode the “lactate clock” to establish a balance between inflammatory damage and anti-inflammatory repair, thereby providing novel targeted intervention strategies for diabetic wound therapy.

## 7 Conclusion

Epigenetic modifications of macrophages, particularly lactylation may be implicated in modulating the transcription and translation of inflammation-related genes in diabetic wounds, which influences the polarization state of macrophages and alters the wound microenvironment through the secretion of cytokines and other factors. Consequently, it modulates the biological behaviors of various cells, including keratinocytes, fibroblasts, and endothelial cells, ultimately affecting the wound healing process. This discovery holds significant clinical importance, highlights lactylation as a potential therapeutic target for mitigating inflammation in diabetic wounds, but the direct causal evidence of the regulatory role of lactylation in diabetic wound inflammation remains limited. Thus, a deeper exploration of the molecular mechanisms by which lactylation regulates macrophage polarization in diabetic wounds contributes to the development of novel therapeutic strategies for diabetes and its complications, particularly chronic wounds.
